# Rainfall Mechanisms for the Dominant Rainfall Mode over Zimbabwe Relative to ENSO and/or IODZM

**DOI:** 10.1100/2012/926310

**Published:** 2012-05-03

**Authors:** Desmond Manatsa, Geoffrey Mukwada

**Affiliations:** ^1^Department of Geography, Bindura University of Science Education, Bindura, Zimbabwe; ^2^Department of Ocean Technology, Policy and Environment, University of Tokyo, Chiba 277-8563, Japan; ^3^International Center for Theoretical Physics (ICTP), 34151 Trieste, Italy; ^4^Department of Geography, University of the Free State, Phuthaditjhaba, South Africa

## Abstract

Zimbabwe's homogeneous precipitation regions are investigated by means of principal component analysis (PCA) with regard to the underlying processes related to ENSO and/or Indian Ocean Dipole zonal mode (IODZM). Station standardized precipitation index rather than direct rainfall values represent the data matrix used in the PCA. The results indicate that the country's rainfall is highly homogeneous and is dominantly described by the first principal mode (PC1). This leading PC can be used to represent the major rainfall patterns affecting the country, both spatially and temporarily. The current practice of subdividing the country into the two seasonal rainfall forecast zones becomes irrelevant. Partial correlation analysis shows that PC1 is linked more to the IODZM than to the traditional ENSO which predominantly demonstrates insignificant association with PC1. The pure IODZM composite is linked to the most intense rainfall suppression mechanisms, while the pure El Niño composite is linked to rainfall enhancing mechanisms.

## 1. Introduction

In southern Africa, precipitation has a dominant influence on national economies, making it a critical variable for study. This is most relevant to Zimbabwe, where large intra-annual and interannual variability in precipitation is evident [1, 2], a condition that is compounded by frequent droughts resulting from climate change [3]. The extraction of subregional dominant modes of rainfall variability, and the identification of associated homogeneous regions, including possible links to particular climate modes, is an essential step in improving seasonal rainfall forecasts, especially during drought and wet years. 

**Figure 1:**
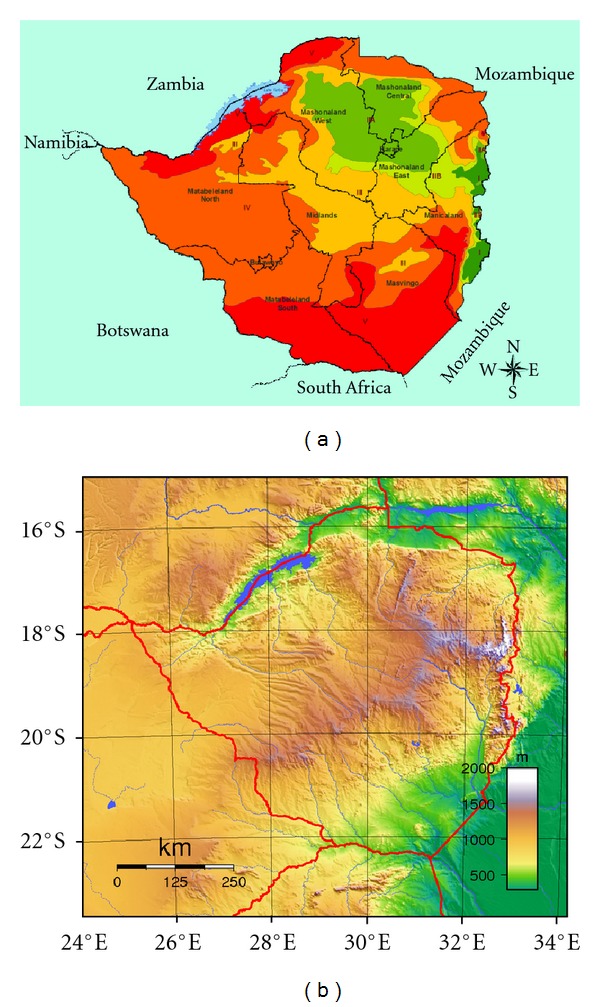
(a) boundaries of agroecological regions I to V of Zimbabwe and (b) the complex nature of relief. Elevated regions cutting diagonally across the country are the Great Dyke Mountains and over the eastern boarder are the Eastern Highlands.

However, Zimbabwe as a country, is physically and climatically complex (Figures 1(a) and 1(b)), making simple comparisons of precipitation characteristics daunting. The country's physiography and atmospheric circulation systems have created a spatially complex precipitation climatology that has resulted in the delimitation of five distinct agroecological regions [4]. These regions are ranked from 1 to 5, according to their suitability to rain-fed agriculture, with 5 being the least suitable. Hence, the demarcation of the regions is mainly governed by rainfall amount, including the interseasonal and intraseasonal rainfall characteristics. Nonetheless, there are several well-recognized climatic controls on precipitation across the subregion that reflect the country's tropical-to-subtropical location, such as the ITCZ and the subtropical anticyclones. These systems imply the existence of nonrandom but well-engineered, coherent synoptic circulation and influence acting on different time scales during the rainfall season. It is, therefore, expected that these structures should in turn control a rainfall pattern of generally uniform variability that is to some extent spatially coherent, especially when averaged over the season. This may explain why the rainfall time series from different stations reveal some improved regularities at monthly-to-seasonal time scales, after having appeared to fluctuate randomly on daily time scales.

Thus based on this revelation, we sought to detect and estimate the interannual deterministic components or “signals” within the rainfall data, in order to characterize the seasonal precipitation variations. But the origins of these variations are uncertain, although there are many studies that try to connect them with different climate forcing factors, such as solar activity and quasibiennial oscillation (QBO) [5], and more popularly known ocean-atmospheric circulation indices, such as ENSO [6–8]. However, several methods exist that make it possible to divide the country's spatiotemporal continuum of precipitation climatology into a manageable number of principal rainfall modes and quasihomogeneous areas. Since the rainfall modes by definition contain rainfall characteristics which covary, they can be linked to different climatic modes.

However, by its nature, precipitation is a very complicated variable [9]. Its description involves a large range of temporal and spatial scales. Thus in the case of Zimbabwe, these attributes can be rendered more acute by the country's orographic patterns (Figure 1(b)), which enhance/suppress some precipitation generating mechanisms. The prevailing north-south orientation of the Eastern Highlands and the northeast-southwest course of the Great Dyke's main mountain chains of the country also play an important role in the spatial distribution of precipitation totals. As a result of the complex terrain, local precipitation amounts are governed by topographic controls at finer scales than the atmospheric controls of overall seasonality and variability.

In order to identify these atmospheric controls, we explicitly avoid relying on the absolute precipitation totals. In this way, the otherwise overwhelming elevation effect is excluded. Hence we adopted instead, the Standardised Precipitation Index (SPI) time series for each rainfall station. The SPI was developed by McKee et al. [10] to quantify and qualify rainfall anomalies. Since this is a standardized quantity, the SPI for the particular station is independent of geographical and topographical differences [11]. The adoption of SPI also renders it possible to make temporal and spatial comparisons which can be readily linked to related atmospheric controls. The other important characteristic of the SPI is that it allows for historical reconstruction of dry and wet periods over a wide spectrum of temporal scales [12], thus enabling the selection of a time scale appropriate to a particular analysis. In this way, different types of droughts can be appreciated using precipitation only, without the need of involving the otherwise complex hydrometeorological variables. Agricultural events, for example, are assumed to be more adequately represented by the SPI of six month time scale. In fact, the SPI at this time scale has already been used to monitor agricultural drought and wet spells in Zimbabwe over multidecadal scales [2].

This study aims to identify spatially coherent precipitation regions based on common patterns of seasonality and variability rather than on simple precipitation totals. It is on these grounds that individual station SPIs become useful for historical reconstruction and analysis of the spatiotemporal cohesiveness of rainfall events. However, to capture the time scales that can be relevant for practical use as in agriculture, the seasonal SPI time scale of six months ranging from October to March was analysed. We employed the principal components analysis (PCA) method to identify the subregional leading modes of SPI variability [13, 14]. This is a useful eigenvector-based technique that enables generalization about areas on the premise of a spatially and temporally varying parameter-like precipitation. The extracted principal SPI modes of variability can then be comparatively connected to the dominant tropically-based climate modes, such as ENSO and the recently discovered IODZM climate mode [11]. In this way, we were able to identify the climate mode with more significant impacts on the leading mode of the rainfall variability, including the associated physical mechanisms.

## 2. Data

The study uses the following data sets: the gridded and station monthly rainfall, the Niño 3.4 and IODZM sea surface temperature (SST) data set, and atmospheric anomalies including the wind and outgoing long-wave radiation.

### 2.1. Station Rainfall

**Figure 2:**
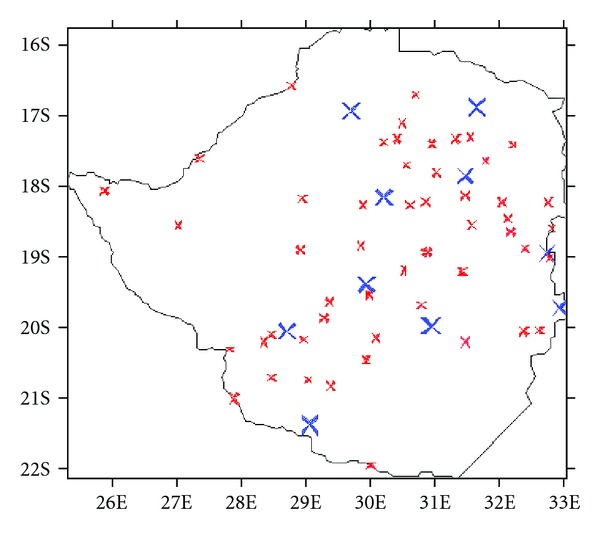
Location of 60 rainfall stations used in the study. Bigger crosses are for stations with more than 100 years of rainfall data.

The Zimbabwe Meteorological Services Department had initially provided time series for 100 stations from its archive. However, some precipitation series suffered from missing data, and some station series were too short. For better PCA results, the time series require long successive records of data and have to be nearly regularly distributed over the country. This is because a PCA performed on unevenly spaced points, like those of precipitation which are highly variable [9], can alter the loading patterns significantly. Another limitation was that some of the stations were located at a considerable distance from the border, and this could have resulted in distorting the loading patterns towards the borders. Thus, to circumvent the perceived “edges” effect resulting from the PCA method, we also gave priority to stations that were located close the country's border. This decision ensured that the rainfall station field was also resolved with respect to proximity to the borders. As such, for the sack of fulfilling the requirements for more robust PCA results which would permit reliable climatic conclusions to be drawn, we eventually settled on a total of only 60 stations (Figure 2).

The selection criteria was based on: (a) length of records with a common continuous period of 1940 to 2000, (b) proximity to the country's border, (c) missing records not exceeding 5% for a station, and (d) distance between stations. This spatial distribution also resolved reasonably well the main topographical units of the country (Figure 1(b)) and hence can adequately project the thrust of the current study. Since this type of analysis demands complete records, missing data were replaced using the arithmetic mean method, details of which can be obtained from WMO [15]. For quality control, the data was tested for homogeneity, using the mass curve analysis [16]. The few nonhomogeneous series identified were corrected.

### 2.2. Gridded Rainfall Data

To analyse the rainfall and the related atmospheric variables from a regional perspective, we demarcated a subregion over southern Africa using the latitudes 25°S to 15°S and longitudes 20°E to 40°E. We referred to this region as east central Southern Africa and assumed that the region can easily resolve atmospheric systems of importance to rainfall variability over Zimbabwe. The rainfall dataset for this region was extracted from high-resolution global monthly total precipitation fields from the University of Delaware. This dataset is composed of 0.5° latitude-longitude grid interpolated rain gauge-based rainfall field that is land only and covers the period 1950–1999. For a complete description of this dataset, see web pages at http://www.esrl.noaa.gov/psd/data/gridded/data.UDel_AirT_Precip.html. Although the reliability of the observed rainfall data over much of the region is questionable, it is considered as the best free and readily available data over the region [17]. Despite providing a wider coverage to approximate the regional rainfall anomalies, this additional gridded dataset also served to verify the reproducibility of the results from a different dataset.

### 2.3. Atmospheric Variables

Atmospheric variables for east central Southern Africa were derived from NCEP/NCAR reanalysis data. The NCEP/NCAR Reanalysis data is a continually updated gridded data set representing the state of the earth's atmosphere, incorporating observations and numerical weather prediction (NWP) model output dating back to 1948. It is a joint product from the National Centers for Environmental Prediction (NCEP) and the National Center for Atmospheric Research (NCAR). We calculated the outgoing long-wave radiation (OLR), zonal, meridional, vector wind, and rainfall anomalies for the pure IODZM, pure El Niño and cooccurrence composites online, using the facilities provided by the website http://www.esrl.noaa.gov/psd/cgi-bin/data/composites/.

### 2.4. ENSO and IODZM SST Indices

ENSO is the dominant climate mode within the tropics, while the supremacy of the IODZM is largely confined to the tropical Indian Ocean [18, 19]. To characterise these two climate modes, we used the classical ENSO and IODZM indices of observed SST anomalies, computed from the Hadley Centre sea ice and SST (HadISST) data set [20]. Thus, the ENSO index is based on the SST anomalies averaged in the Niño 3.4 region (170°W–120°W; 5°S–5°N), while the IODZMI index is based on the SST anomalies in (50°E–70°E; 10°S–10°N) minus those in (90°E; 110°E; 10°S; 0°N). Both indices are normalized relative to their standard deviations. The HadISST SSTs were derived from the website: http://climexp.knmi.nl/. The strength of ENSO is taken as the average of November to January SST anomalies, which is the period when the ENSO influence has been found to be strongest on the seasonal summer rainfall [21]. The IODZM index is calculated from the average of the most active months of September to November (SON; [11]). Events exceeding 1 standard deviation were considered extreme events. Using this criteria, we extracted 1950, 1961, 1967, and 1983 as pure IODZM events; 1951, 1957, 1968, and 1976 as pure El Niño events; 1963, 1972, 1977, 1982, 1991, 1994, and 1997 as cooccurrence events.

## 3. Methods

In addition to composite and partial correlation analysis, the study relies heavily on linear statistical tools in the analysis of the rainfall and SST time series. This is because the SSTs and rainfall time series have been found to be largely normal, making linear assumptions in relationships reasonable.

### 3.1. The Standardised Precipitation Index Method (SPI)

The SPI method was used, as it is capable of retaining fundamental parameters in the analysis of the occurrence of different events of desired scale in terms of severity, magnitude, and frequency, irrespective of topographical influences. Technically, the SPI represents the total difference of precipitation for a given period of time (e.g., six-month period for this study) from its climatological mean value normalized by the standard deviation for the same six months in the climatological annual cycle, computed over the entire period of analysis [22]. In this way, dry (wet) events are represented by negative (positive) SPIs. The 6-month time scale SPI (October to March) has been chosen because it covers the critical months for agriculture in Zimbabwe.

The country's standardized precipitation index (Zim SPI) was calculated by aerially averaging all the stations monthly rainfall before computing the 6-month scale SPI values. The near even distribution of the stations over the country made it possible to achieve a spatially balanced index. The SPI values were calculated online using facilities provided from the website http://drought.unl.edu/MonitoringTools/DownloadableSPIProgram.aspx. Details about the SPI computation can be found in several papers including McKee et al. [10], Guttman [23], and Vicente-Serrano [24].

### 3.2. Principal Component Analysis (PCA) Method

The PCA method is a widely known statistical technique, used very often in meteorology and climatology. Basically, the PCA technique reduces information in a large number of variables into a smaller set, while losing only a small amount of information. This reduction in dimensionality can lead to a more tractable understanding and interpretation of multivariate data in the form of different modes of variability within the dataset. In this study, PCA was used to describe the station SPI time series data set by means of new few variables (principal components, (PCs)), which have two fundamental properties: (a) two different components that are uncorrelated, (b) each component is derived from an empirical orthogonal variable, accounting for a maximum in residual total variance of the SPI data set. The purpose of using the PCA was to identify the most important correlation structures between the station SPIs within the study domain in order to obtain a description of the major part of the overall variance with few linear combinations based on the SPI variables. By capturing patterns of SPI covariability countrywide, we should be able to demonstrate that the station SPI time series shows a more or less singular tendency that can then be related to the dominant climate modes of ENSO and IODZM. Details of this PCA method including the interpretation of its spatial and temporal modes can be obtained in several papers and most standard advanced statistics texts [25–27].

### 3.3. PCA Using Station SPI Time Series

The use of the SPI method which by its nature initially eliminates possible scale effects as opposed to the station's total seasonal rainfall was first proposed by Bordi and Sutera [13]. Standardizing rainfall across the country's domain gives equal weighting to all stations, and hence interactions between regions of differing total rainfall could be identified. It therefore allows dry stations in regions of relatively low rainfall in the southern parts of the country (regions 4 and 5) to be directly compared to relatively wet stations in the Eastern Highlands (agroecological regions 1 and 2, Figures 1(a) and 1(b)). With the dual exploitation of the SPI and PCA methods, we expect stations which have similar seasonal timing of SPI to be interlinked and grouped together, presumably because of the same atmospheric controls, even if absolute precipitation amounts differ simply because of the elevation effect. In this case, the PCs become a new set of a manageable number of orthogonal variables that contain condensed information expressed from the SPI field. The spatial presentation of each PC loading is achieved through extracting corresponding values of each station and mapping them through contour plotting using SURFER [28] software (e.g., Figures 4(a)–4(d)). However, as with any statistical tool, there is no guarantee that these evaluated factors represent dynamically existing entities within the country's rainfall system. Thus, it is important to determine whether or not these results have any physical meaning. The basic assumption is that the underlying PCA dimensions or factors can be used to explain complex meteorological or other climatological variables.

### 3.4. Partial Correlation

To show the independent influence of the IODZM/ENSO on seasonal rainfall, the partial correlation technique was employed. In this approach, the exclusive relationship between two variables can be realized while excluding the influence arising from another independent variable. The partial correlation between Zim SPI and IODZMI, while excluding the relation arrived at because of the correlation between ENSO and Zim SPI, is defined as follows:

(1) $$\begin{array}{cc} & r_{\text{zimspi \& iodzmi, nino3.4}}\\ & \;\;=\frac{r_{\text{zimspi \& iodzmi}}-r_{\text{zimspi \& nino3.4}}\times r_{\text{nino3.4 \& iodzmi}}}{\sqrt{1-r_{\text{zimspi \& nino3.4}}^{2}}\sqrt{1-r_{\text{nino3.4 \& iodzmi}}^{2}}}, \end{array}$$

where *r*_zimspi  &  iodzmi_ is the correlation between Zim SPI and IODZMI, *r*_zimspi  &  nino3.4_ is the correlation between Zim SPI and Niño 3.4 index, and *r*_nino3.4  &  iodzmi_ is the correlation between Zim SPI and IODZMI. 

Similarly, the partial correlation is obtained for Niño 3.4 and Zim SPI while excluding the influence due to the correlation between IODZM and Zim SPI. Although there are a number of limitations to this approach, most notably related to linearity, it does provide a basic way of analysing variability of ENSO (IODZM) independent of IODZM (ENSO). This approach was used in a number of previous studies for the same purpose (e.g., [18, 19, 29]).

In addition to the length of the indices, the statistical significance of cross-correlations be it partial or simple, also largely depends on the autocorrelation characteristics and smoothing of each time series involved. In this study, there is no prior smoothing on all time series used in the cross-correlation coefficients presented. Time filters were not applied to any of the above data sets to remove periodic variations. The coefficients were also based on yearly sampled series (i.e., the time interval between two observations is one year), thus showing insignificant lag-1 autocorrelation [11]. To evaluate the statistical significance of the correlation coefficients, the standard two-tailed Student's *t*-test was used.

## 4. Results

### 4.1. The Spatio-Temporal Modes of the Dominant PCs

**Figure 3:**
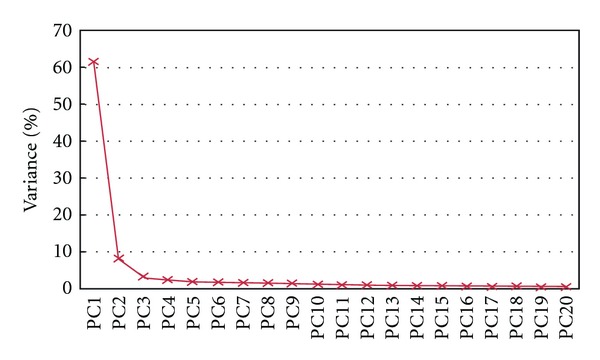
Principal components' scree plot. Only the first 20 PCs of the resulting 70 PCs are shown.

One of the objectives of performing PCA was to identify the main physical mechanisms of rainfall enhancement and suppression covariability patterns of the seasonal SPI field. These can then be used to explain the most important rainfall contrasts, as illustrated by the PCs. Although several criteria have been suggested for deciding how many PCs to retain in order to differentiate “signal” from “noise” [30, 31], a clear-cut number of PCs is still debatable. In this study, we adopted the simple scree test method of Cattell [32] that uses a scree plot. Results for the scree test method to determine the point of truncation of significant PCs are shown in Figure 3, where we selected for presentation only the first 20 of the resulting 60 PCs.

By means of this dominant-variance selection method, it can be noted that a more conspicuous break of the slope occurs at the forth PC. This can be interpreted as implying that the first four of the 60 eigenvalues are significantly different from noise. Therefore, these four PCs were considered worth retaining for further analysis. Moreover, the level of explained variance of 75.72% (Table 1) by the retained PCs seems to be acceptable, given the considerable number of rainfall stations and the complex nature of the rainfall variable at the seasonal scale. It then follows that only maps constructed from loadings for PCs 1–4 are assumed to comprise subtle variations of pattern that can easily be associated with known modes of rainfall development. Thus, in this paper, the PCA is utilized not only as a data reduction technique, but also as a method which ensures that only the fundamental modes of station SPI variation are considered.

### 4.2. The Spatial Mode

**Figure 4:**
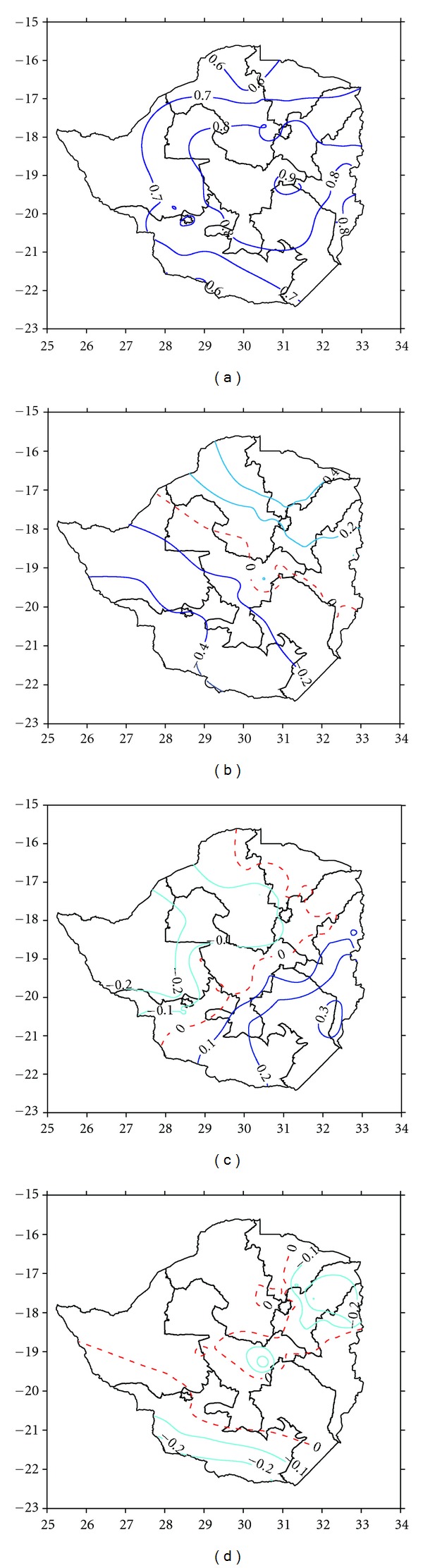
The spatial distribution of loading patterns of the (a) first, (b) second, (c) third, and (d) fourth PC of the 6-month SPI for the month of March. The zero isopleth is depicted by the broken red line, while positive and negative zones are indicated by dark and light blue isopleths.

It is interesting to note that all the four PC plots (Figures 4(a)–4(d)) do not exclusively distinguish the Eastern Highlands as a distinct rainfall region, although it has on average the highest rainfall over the country. Neither do they extract the lower elevation southern regions as a unique area of low rainfall. It should be noted that interpreting the spatial nature of these different PCs, although appearing simple in concept, does not seem to be the choice that best preserves the underlying physics of precipitation generation and suppression. Thus, our approach ensures that the relief systems that largely determine the rainfall quantities are not significantly separated from the rest of the country in both time and space.

PC1, which is the first source of variance in the data is associated with the overall magnitude of seasonally averaged rainfall events in the country and explains more than 61.69% of the total variance. We see that the PC1 plot (Figure 4(a)) is unidirectional and centered, being characterized by relatively high values (>+0.6) of factor loadings that cover more than 95% of the country. This indicates high positive and spatially homogeneous correlation between drought/wet events variability at the individual stations and PC1. Effectively, there is no distinct contrasting internal structure within the country as far as the 60 points are able to represent this homogeneity. Accordingly, the station SPIs are highly homogeneous, with space-time variability being relatively small, and hence it can be reasoned that PC1 may represent the dominant rainfall mode of variability associated with the countrywide station SPI field. It is therefore most likely that a severe wet/drought seasonal event may simultaneously cut across a substantial portion of the country. Hence, a single aerially averaged SPI time series like the Zim SPI, may be good enough to represent individual station drought/wet seasonal event variation across the country.

The combined effort of the other three PCs can hardly explain 14% of the total variance, meaning that they individually explain relatively small fractions of the total variance. From Table 1, we see that from PC2 to PC3 explain 8.2%, 3.4%, and 2.4% of the total variance, respectively. Their corresponding spatial representations do not only display relatively small magnitudes of PC loadings, but also exhibit opposing signs between the different zones. The complexity exhibited by their spatial structures increases, as their PC number grows (Figures 4(b)–4(d)). However, it has to be noted that on the whole, the magnitudes of these three PC loadings are too small to provide for a much clearer interpretation. Their spatial loadings, especially those for PC3 and PC4, are not significant enough (even at 90% confidence level) to derive any plausible conclusion from their analysis.

### 4.3. The Temporal Mode

The corresponding casewise scores are analysed in order to illustrate the extent to which the four extracted PCs are able to represent Zim SPI in the national drought/wet seasonal event analysis. These PC time coefficients are useful in identifying seasonal events that are peculiar to the temporal patterns of each PC. Table 1 shows the percentage of the total variance attributed to each EOF as well as the correlation between time series of the associated amplitude coefficients or PCs and the aerially averaged Zim SPI. Judging from the correlation coefficients shown in this table, it should be noted that it is only the resulting time series of EOF coefficients associated with the leading PC that are significantly related to the Zim SPI time series.

**Figure 5:**
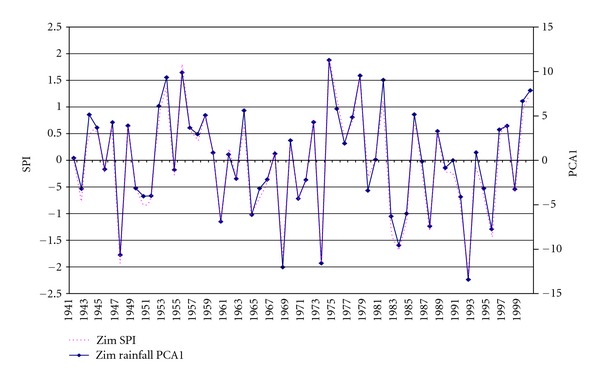
Time series of PC1 alongside Zim SPI for the period from 1941 to 2000.

As shown in Table 1, PC1 does not only have a near perfect positive connection with Zim SPI, but also is the only PC with a statistically significant relationship. The fact that the correlation between the two time series is 0.992 illustrates an exceptionally strong association between PC1 and Zim SPI. Thus, to further demonstrate this important aspect, we show in Figure 5 the temporal manifestation of PC1 alongside with the Zim SPI. In this figure, it is evident that the amplitudes of PC1 time series have a very strong direct incident on the magnitude of the country's rainfall extremes. They confirm a remarkably good representation of the seasonal rainfall variability, in particular capturing virtually all the strong and weak (drought/wet) years of the rainfall between 1941 and 2000. Therefore, PC1 is the consistent mode of the seasonal variation of the country's rainfall, strongly suggesting its dominance and robustness in representing the long-term drought/wet seasonal event variability in Zimbabwe.

Therefore, it can be reasoned that the sum of the underlying physical rainfall triggering mechanisms at national level is best represented by PC1. In view of this manifestation, our attention will now focus exclusively on PC1. We consider this dominant rainfall mode to sufficiently represent the Zim SPI and not the other modes of variability. Hence, the country's dominant rainfall anomalies form a spatial and temporal pattern that typically varies concurrently as part of one principal component making. This confirms that a relatively strong spatial and temporal station drought/wet seasonal event relationship prevails. Therefore, Zim SPI is most likely to contribute a fairly good representation of the dominant rainfall mode related to the extreme rainfall that constitutes the regional characteristic development of rainfall excess/deficits.

**Figure 6:**
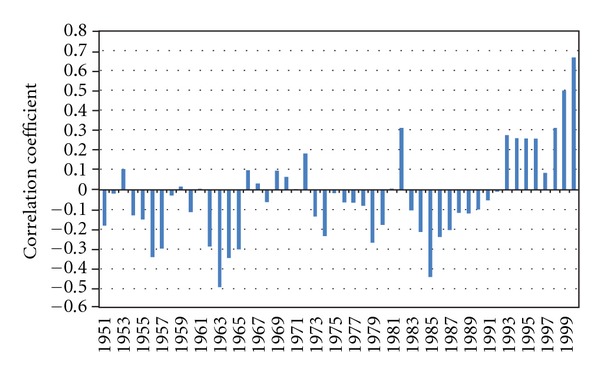
Ten-year running correlation between Zim SPI and PC2 for the period from 1951 to 2000.

Further investigations using a ten-year overlapping moving correlation between PC2 and Zim SPI showed predominantly negative coefficient values. This principally negative association later reversed to become consistently positive from 1992 (Figure 6) and reached significant levels above the 90% confidence level from 1997. This is surprising, considering that the overall correlation with Zim SPI, when the entire study period is considered, is very small (0.068) and insignificant. The link of the Zim SPI to the other two PCs, whether the correlation is taken on smaller periods of running segments or applied to the whole period of analysis, remains insignificant. The fact that PC2 became significantly in phase with Zim SPI after 1997 means that there was a gradual transmission in the seasonal rainfall system's components responsible for the properties represented by PC2, as they approached a national character (Zim SPI) after 1997. This is a novel subject for further research.

### 4.4. ENSO/IODZM Association with the Dominant Mode of Rainfall Variability

In order to determine possible large-scale forcing of the observed modes of rainfall variability, we chose to link the four PCs with the dominant tropically based atmospheric modes, ENSO and IODZM. These two atmospheric modes were identified as being the most likely large-scale predictors of the country's extreme rainfall events. ENSO is the well-known predictor for southern African rainfall [8]. The IODZM was recently discovered by Saji et al. [11] and is also believed to influence the regional rainfall [21].

In light of this knowledge, we sought to establish how these two climatic modes relate to the four different PCs and then determine the atmospheric mode that is paramount in its impact on specific PCs. The aim of this preliminary PC screening exercise was to assess which PCs were generally significantly influenced by the two climate modes. The nature of the relations was established by initially using a simple cross-correlation method on all the variables used in this study. Since all correlations were calculated between concurrent predictor-predictand observations, we use the term “predictor” not to imply that we would expect any skill with a lag correlation, but rather to imply that these variables are statistically linked to the extreme wet or dry events.

Results of how the four PCs are related to ENSO and IODZM are shown in Table 2. However, for those that were insignificantly associated, no attempt was made to search for other individual predictors that could be significantly linked to these PCs. Attention for investigation was again narrowed to the two predictors' relationship with only the leading PC. This is against the background that relatively high portion of the explained total variance (about 62%) of the factor loadings structure is largely uniform and defined by the same principal component (PC1). This could provide a physically explainable mechanism dominantly affecting nationwide extreme dry or wet conditions. Establishment of the more dominant impact between ENSO and IODM on PC1 became the main focus of the subsequent sections. 

**Figure 7:**
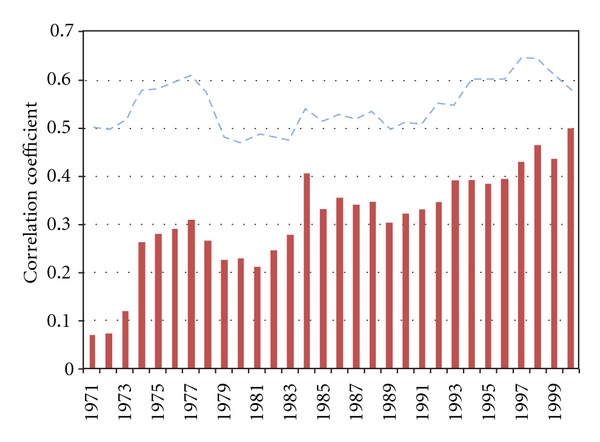
Temporal manifestations of 31-year overlapping segments of correlation coefficients between PC1 and ENSO (solid bars); PC1 and IODZM (broken line).

When the relationship presented in Table 2 is subjected to climatological period (31 yrs) overlapping segments, it is revealed that the ENSO association with PC1 became consistently significant above the 95% confidence level only after the 1980s and exceeds the 99% level from 1997 (Figure 7). On the other hand, the connection with IODZM has been consistently well above this confidence level (above 99%) throughout. Both Niño 3.4 and IODZMI are insignificantly related to the rest of the PCs (PC2 to PC4). This shows that these two atmospheric modes may not be associated with any significant linear control over these modes of rainfall variability. 

The above analysis could have been more appropriate if ENSO and IODZM were independent climate modes in their respective oceans. The reality is that ENSO and IODZM interfere with each other at times [18, 33]. This acknowledgement rendered the earlier employed method of analysis unsuitable. Unfortunately, this simple correlation analysis has been traditionally used for the region in previous paper to detect the ENSO signal on regional rainfall variability [6–8]. The reason could be the late discovery of the existence of other competing climate modes of close proximity, that cooccur and interact with ENSO at times, like the IODZM. It is prudent to employ the partial correlation technique in order to extract the assumed statistically independent influences of each climate mode. Using this technique, it is possible to get the partial correlation between IODZM (ENSO) and the rainfall, while excluding the relation arrived at because of the influence of ENSO (IODZM). This method has been successfully used in several researches with the same goal [18, 33]. 

**Figure 8:**
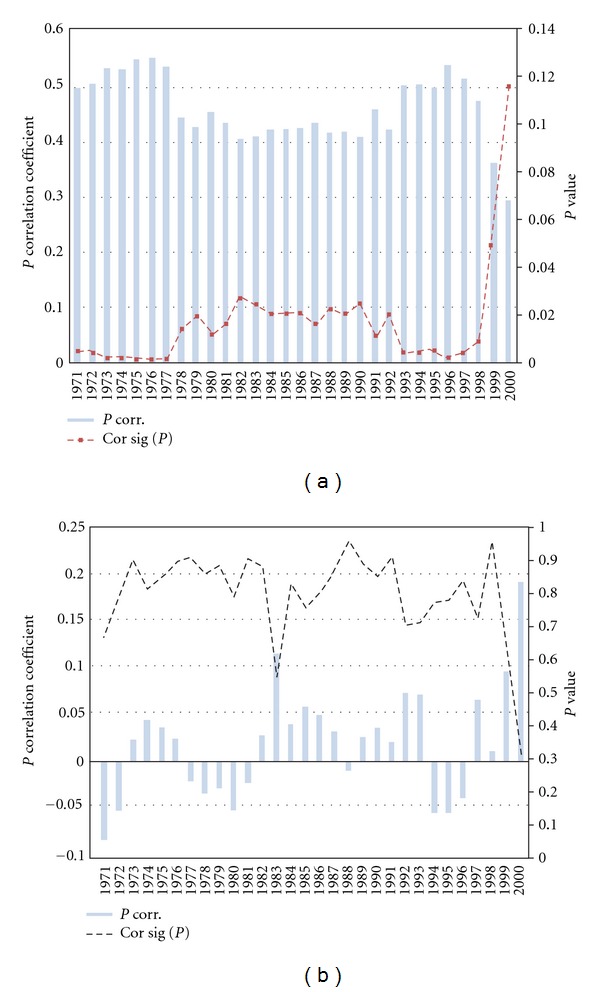
Moving partial correlation between (a) Zim SPI and IODZMI when Niño 3.4 signal is removed; (b) Zim SPI and Niño 3.4 when IODZMI signal is removed (bars) alongside corresponding running *P* values (broken lines). Partial correlation vales have been multiplied by −1 for ease of comparison. Values are plotted as a function of the end year in the 31-year interval.

Figures 8(a) and 8(b) displays the results for the 31-year moving partial correlation analysis performed between PC1 and IODZMI (ENSO), when ENSO (IODZMI) influence was statistically suppressed. The less important role of ENSO is demonstrated not only through the stronger and more stable temporal relationship of IODZMI with PC1 (Figure 7), but through the partial relationship as well (Figures 8(a) and 8(b)). In the partial correlation between IODZMI and PC1, the level of significance remains predominantly well above the 95% confidence mark when ENSO signal is removed. This partial relationship largely collapses to near decorrelation when the IODZM signal is removed. It is surprising though, to see that ENSO appears not to play any significant role in the occurrence of drought/wet events over Zimbabwe, especially those events related to PC1. Although the mean correlation of PC1 and ENSO over the full record is negative, this relationship without the IODZMI signal is reversed to positive. Generally, El Niño without IODZM influence is related to above normal rainfall. Thus, it was possible for Goddard and Graham [34] using GCM experiments, to link El Niño events to rainfall surplus rather than deficits, contrary to what is widely believed. 

It is, however, interesting to note that for the period after 1996 the *P* values change swiftly to the opposite direction for both ENSO and IODZM correlations with PC1. For ENSO partial correlations, the relationship strengthens dramatically from a *P* value of above 95% to below 32% in 2000, while that of IODZM drastically weakens with *P* values rising sharply from below 1% to about 12%. From 1997, ENSO's influence over the country strengthened at the expense of the weakening IODZM influence [35]. This observation makes it reasonable to link ENSO influence to PC2. This is because ENSO became significantly related to Zim SPI when PC2 became strongly in phase with Zim SPI after 1997. Therefore, it is reasonable to suggest that the decoupling of IODZM from PC1 paved way for the coupling of ENSO to Zim SPI, through PC2. However, we will not discuss these connections further as they fall outside the scope of ongoing research. 

### 4.5. Atmospheric Anomalies Linked to ENSO and/or IODZM Impacts on Rainfall

Since rainfall amounts are relevant to agriculture and other water uses, we revert to analysing the actual rainfall anomalies instead of the SPI. Thus we quantify the effects of pure El Niño, pure IOD, and their cooccurrence in terms of precipitation amounts using the global-gridded rainfall data from University of Delaware. This is largely for the purpose of showing that similar conclusions can be drawn on the country's rainfall anomalies, irrespective of the data set used. The amount of rainfall over the country is largely determined by the moisture contained in the surface wind which is represented by vector wind at the 850 mb level (lower levels). This level has been chosen as it approximates better the pressure pattern that influences the wind at the surface as it irons out the effects of relief and friction. The conditions prevailing in the middle levels are represented by vector wind at the 500 mb level (middle levels). That is the level where rain bearing clouds generally develop. Details of the rain triggering mechanisms over the country can be found in the Climate Handbook of Zimbabwe [36]. 

**Figure 9:**
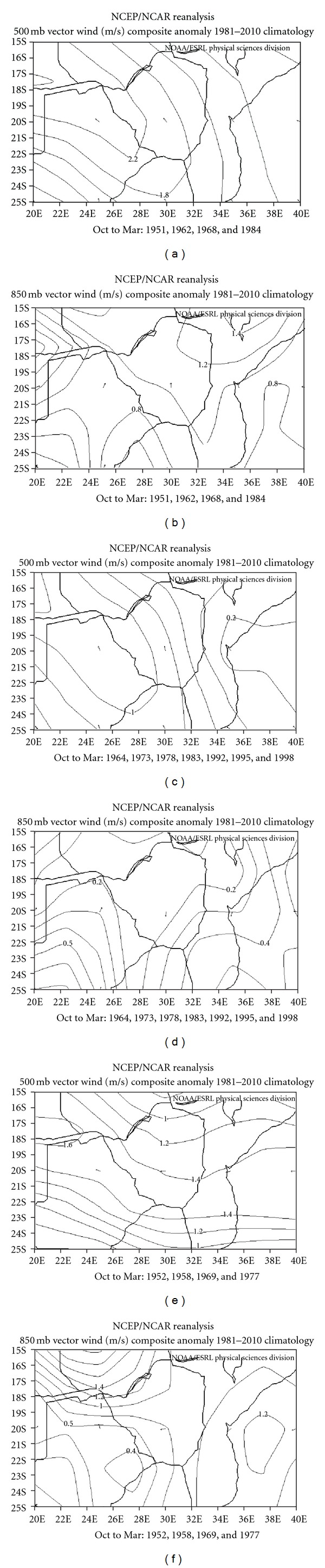
Vector wind pattern (m/s) for the 500 mb and 850 mb pressure level for (a) and (b) pure IODZM composite, (c) and (d) cooccurrence composite, and (e) and (f) pure El Niño composite over the east-central southern African region.

In this section, we analyse the scalar winds at the lower levels and the middle levels (Figure 9) so as to document their characteristics during the pure El Niño (a, b), pure IODZM (e, f), and cooccurrence composites (c, d). This enables better understating of how these events influence the pressure patterns that drive the winds at the two levels which result in rainfall anomalies (largely PC1) peculiar to each composite. Figure 9 shows vector wind anomalies at the surface (left) and middle levels (right) for the pure IODZM, pure El Niño, and cooccurrence composites. The pure IODZM composite for the middle levels shows a large anticyclonic circulation over the region. The strongest circulation anomalies are over the west, where the vector wind anomalies exceed 3 m/s. Since this is the level where the rain bearing clouds develop in depth, the anticyclonic anomalies limit vigorous cloud formation because of its tendency to induce subsidence. The anomaly winds at 850 mb level are equally unfavourable for cloud formation with relatively strong anomalies that exceed 2 m/s. These winds are south-westerly over the south with a nearly closed anticyclonic circulation over the north, making the anomaly airflow over the country have a predominant continental origin. 

For the cooccurrence composite, the anticyclonic anomaly still lies over the region in the middle levels, but is less intense with maximum vector wind anomalies less than half of those displayed by the pure IODZM composite (1.3 m/s). There is also a cyclonic anomaly over the east of the country in the west of the Mozambique Channel. This further reduces the anticyclonic rainfall suppression ability over the eastern part of the country. At the 850 mb level, the winds are not very anomalous, with maximum vector wind speeds less than 0.3 m/s, compared to 2 m/s for the pure composite. The anomalous direction is mostly north-easterly, which gives the anomalous wind some limited moisture fetch from the lower latitudes of the tropical Indian Ocean. This indicates that the circulation is not as conducive to severe rainfall deficits as in the pure IODZM composite. 

The most interesting circulation anomaly is found in the pure El Niño composite. Here, the circulation is almost the opposite of the other two composites, especially the pure composite. A cyclonic wind anomaly sits where an anticyclonic wind anomaly previously rested in the other two composites, to the west of the country. The vector wind anomaly is predominantly easterly over the country with maximum wind anomalies near the centre of the middle level anomalous low-pressure system. The surface wind anomalies are even more favourable. Anticyclonic circulation anomaly over south east Botswana drives an south easterly anomaly, whilst a cyclonic anomaly over south west Zambia forces a north-westerly wind anomaly over the country. This scenario is conducive for convergence over the country, whereby the moist and warmer air from the Congo Basin to the north is undercut right over the country by the more stable and cooler airflow from the subtropical regions to the south. 

**Figure 10:**
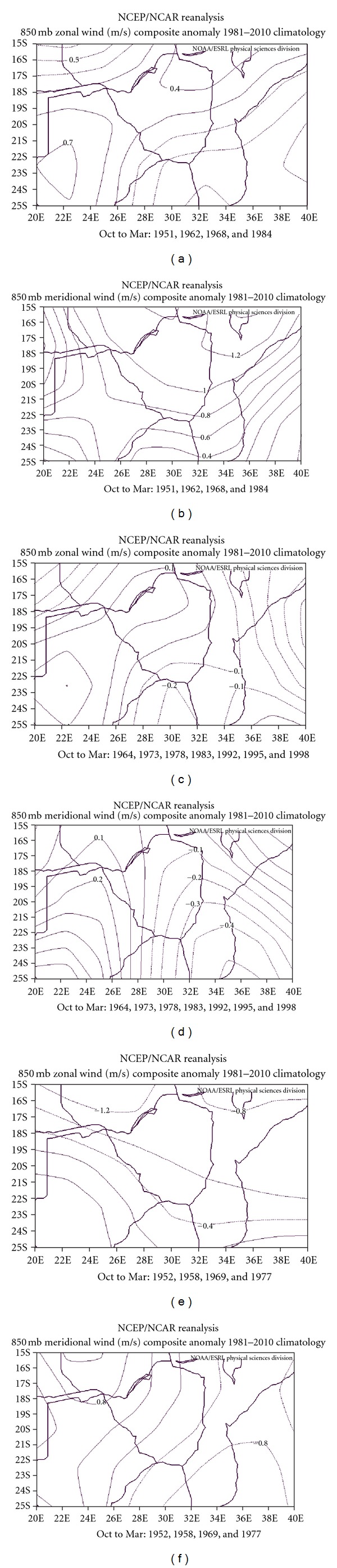
Zonal (left) and meridional (right) wind pattern (m/s) for the 850 mb pressure level for (a) and (b) pure IODZM composite, (c) and (d) cooccurrence composite, and (e) and (f) pure El Niño composite over the east-central southern African region.

To get a clearer picture of these important characteristics of the surface winds to rainfall anomaly development, the 850 mb vector winds are resolved into their zonal and meridional components. Thus, in Figures 10(a)–10(f), we show the zonal (left) and the the three composites. The pure IODZM composite shows meridional (right) wind anomalies at the 850 mb level for predominantly westerly anomaly with winds exceeding 1.5 m/s (Figure 10(a)). The meridional wind anomalies have a relatively strong positive anomaly throughout the country; with maximum speeds exceeding 1.2 m/s (Figure 10(b)). This means that the relatively strong westerly components have the tendency of fetching dry air from the largely desert land of Botswana to the west. The relatively strong southerly component (possibly resulting from enhanced subtropical high-pressure cell to the south) has the ability to displace any possible anomalous zone of convergence well to the north, out of the country. The wind component anomalies for the cooccurrence composite are not very anomalous. The zonal wind anomalies have both positive and negative values of small magnitudes ranging from slightly −0.5 m/s north of 20°S to just above 0.5 m/s, south of this latitude. On the other hand, the meridional anomalies have some negative values throughout with minimum values lower than −0.3 m/s. Whilst no significant deduction can be derived from the zonal component, the stronger northerly component entails the suppression of the convergence zone over the country during this composite. The zonal component of the pure El Niño composite has a relatively strong and predominantly easterly component that exceeds −1.0 m/s to the northwest. The meridional components have both northerly and southerly components which are relatively strong over the country. The relatively strong easterly components entail a fetch of moisture from the tropical Indian Ocean, while the opposing meridional components ensure that convergence of the tropical and subtropical airmass occurs over the country. 

**Figure 11:**
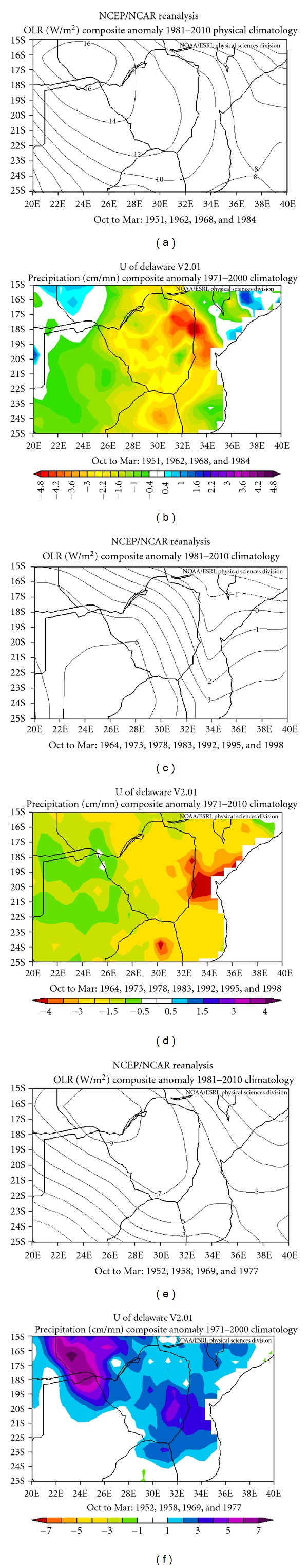
OLR (left) and precipitation anomaly patterns for (a) and (b) pure IODZM composite, (c) and (d) cooccurrence composite, and (e) and (f) pure El Niño composite over the east-central southern African region.

The resulting impacts on rainfall anomalies from the anomalous wind (pressure) systems are shown in Figure 11. In this figure, the outgoing long-wave radiation (OLR), which is proxy to deep convection (left) is shown alongside the actual daily rainfall anomalies (right) for each composite. Figures 11(a) and 11(b) shows the pure IODZM composite. Despite the general rainfall suppression mechanisms that can be implied, it is noted that the dependence of rainfall total on relief which had previously been suppressed by the use of the SPI reappears. We note that although the strongest positive OLR anomalies coincide with the region where the intensity of the middle level anticyclone was strongest, the rainfall deficits are more pronounced over the regions, cutting diagonally from the north-east to the south-west (location of the Great Dyke) and the north of the Eastern Highlands. In other words, the precipitation deficits are notably greater over the regions of higher relief (compare with Figure 1(b)). Since the wind over the country will be drier during these events, the amount of rainfall to potentially result from the airflow during relief-aided uplift will be greatly curtailed. It then follows that the rainfall deficits are more pronounced in areas where the relief could have had the capacity to potentially trigger more rainfall should the airflow had been moister. 

The same applies to the cooccurrence composite (Figures 11(c) and 11(d)), though it has less intense positive OLR anomalies and rainfall deficits. Consistent with earlier analysis, the pure El Niño composite shows the near opposite of the pure IODZM composite. Negative OLR values are found throughout the region, with the lowest anomalous centre located where the middle level low-pressure system was positioned. In this composite, the strongest rainfall surpluses coincide with the centre region of middle level low-pressure anomalies. The elevated regions of the eastern border highlands and the Great Dyke also portray areas of significantly high-rainfall anomalies. 

## 5. Conclusions and Recommendations 

This paper has presented Zimbabwe's dominant modes of precipitation variability by means of PCA's temporal and spatial modes, using the SPI rather than direct rainfall values. The SPI suppresses variations in rainfall that are sympathetic to altitude while enhancing the homogeneity of the true rainfall triggering system signals. Using this approach, it was possible to demonstrate that the leading PC (PC1) is by far the most dominant mode of the country's rainfall variability and can be used to represent the major rainfall patterns in the country, both spatially and temporarily. The other modes have been shown to be insignificant. This observation provides a better understanding of the complex atmospheric phenomena that are responsible for the general seasonal rainfall distribution over Zimbabwe. The sum of the underlying physical processes that are responsible for the emergence of the homogeneous precipitation region represented by PC1 is highly correlated with Zim SPI. Thus, it may not be recommended to subdivide the country into the two seasonal rainfall forecast zones, as is the current practice. The difference between the two zones, which seem to have been largely determined by the human perception of considering relief and rainfall amount in differentiating homogeneous regions, was rendered insignificant in this study. 

ENSO has been traditionally attributed to the major controls of the underlying regional precipitation-generating processes. It is of interest to note that for the period under review, it is the IODZM influence, rather than ENSO, which had the paramount influence on the country's dominant mode of rainfall variability (PC1). The pure IODZM composite is associated with the most severe rainfall deficits, more pronounced over the high altitudes of the country. The composite for cooccurrence events is associated with relatively less severe rainfall deficits and seem not to be particularly conditioned by elevation. It is noted that the pure El Niño composite is associated with rainfall surplus quantities which are not only strongly defined by elevation but by the location of the anomalous center of the middle level low-pressure system as well. This is not the first time that this observation has been documented. Using GCM experiments, Goddard and Graham [34] linked El Niño events to rainfall surplus, rather than deficits, contrary to what is widely believed. Thus, the IODZM could have provided better and more consistent seasonal rainfall prediction skill for the country than the traditional predictor, ENSO. However, it is speculated that the IODZM became decoupled from the Zim SPI (PC1) since 1997 [35]. 

In addition to the findings of this research, further evidence will be necessary about precipitation events during the dominance of one specific weather type. It is also obvious that in reality, atmospheric processes cannot by their nature be decomposed into orthogonal (i.e., uncorrelated) processes, as has been necessarily done by the use of the PCA in the current paper. Hence, there is no rationalization that the orthogonal variables must represent physically occurring processes as these variables impose great restrictions on mathematical solutions. In reality, causal mechanisms of precipitation are fuzzy and overlapping, and this aspect could have been difficult to capture in the PCA technique. Besides these shortcomings, the reduction of the information given by the rainfall records of 60 point SPIs to an exceedingly leading single PC, with just a loss of about a third of the total variance will be of great utility if the PC time series instead of the complete original records are incorporated in regional climatological and/or hydrological models. 

## Figures and Tables

**Table 1:** Eigenvalues, percentages of the total variance explained by EOF 1 to 4, the corresponding percentage of the accumulated variance, and the correlation values between the PC amplitudes and Zim SPI. Significant values above 95% confidence level are indicated in bold.

EOF	Eigenvalues	Variance percent	Accumulated variance percent	Correlation Coeff. with Zim SPI
PC1	33.835	61.686	61.686	** 0.992**
PC2	4.521	8.243	69.929	−0.068
PC3	1.867	3.405	73.333	−0.021
PC4	1.31	2.388	75.721	0.026

**Table 2:** Contingency table for the cross-correlation among variables used in the study.

	Zim SPI	PC1	PC2	PC3	PC4	Niño 3.4	IODZMI
Zim SPI	1	0.992	−0.068	−0.021	0.026	−0.402	−0.480
PC1		1	0	0	0	−0.408	−0.484
PC2			1	0	0	−0.141	−0.040
PC3				1	0	−0.099	−0.174
PC4					1	−0.134	0.116
Niño 3.4						1	0.584
IODZMI							1
